# Range size positively correlates with temperature and precipitation niche breadths but not with dietary niche breadth in triatomine insects, vectors of Chagas disease

**DOI:** 10.1371/journal.pntd.0012430

**Published:** 2024-08-16

**Authors:** Fernanda S. Caron, Daniel Rivadeneira, Jorge Rabinovich, Marcio R. Pie, Juliano Morimoto

**Affiliations:** 1 Programa de Pós-graduação em Ecologia e Conservação, Universidade Federal do Paraná, Curitiba, Brazil; 2 Programa de Pós-graduação em Zoologia, Universidade Federal do Paraná, Curitiba, Brazil; 3 Centro de Estudios Parasitológicos y de Vectores (CEPAVE CONICET-CCT La Plata, Universidad Nacional de La Plata), La Plata, Province of Buenos Aires, Argentina; 4 Department of Biology, Edge Hill University, Ormskirk, Lancashire, United Kingdom; 5 Institute of Mathematics, University of Aberdeen, King’s College, Aberdeen, United Kingdom; Instituto Pasteur de São Paulo, BRAZIL

## Abstract

Ecological theory predicts that species that can utilise a greater diversity of resources and, therefore, have wider niche breadths should also occupy larger geographic areas (the ‘niche breadth-range size hypothesis’). Here, we tested this hypothesis for a blood-sucking group of insects of medical significance: the Triatominae (aka ‘kissing bugs’) (Hemiptera: Reduviidae). Given that niches can be viewed from different perspectives, we tested this hypothesis based on both dietary and climatic niches. We assembled the most complete dataset of triatomine feeding patterns to date by reviewing 143 studies from the literature up to 2021 and tested whether the niche breadth-range size hypothesis held for this group for both dietary and climatic components of the niche. Temperature and precipitation niche breadths were estimated from macro-environmental variables, while diet breadth was calculated based on literature data that used PCR and/or ELISA to identify different types of hosts as blood sources per triatomine species. Our results showed that temperature and precipitation niche breadths, but not dietary breadth, were positively correlated with range sizes, independent of evolutionary history among species. These findings support the predictions from the range size-niche breadth hypothesis concerning climate but not diet, in Triatominae. It also shows that support for the niche breadth-range size hypothesis is dependent upon the niche axis under consideration, which can explain the mixed support for this hypothesis in the ecological literature.

## Author summary

Many triatomine species carry the parasite *Trypanosoma cruzi*, a flagellate protozoan that causes Chagas disease in animals and humans, posing a long-standing public health threat to communities in the Global South. We investigated how the niche of triatominae species correlates with their geographic range. We compiled a comprehensive dataset on their feeding habits and environmental preferences and found that temperature and precipitation niche breadth correlated geographic range size but dietary breadths did not. These findings highlight the complexity of ecological relationships and contribute to a better understanding of how species interact with their environment, and can play a crucial role in determining the range of these vectors. Thus, this knowledge can aid in the development of more effective strategies for managing vector-borne diseases.

## 1. Introduction

A long-standing hypothesis in ecology is that species that can utilise a greater array of resources (‘niche breadth’) should occupy larger geographical ranges [1,2]. As a result, a positive correlation between niche breadth and range size is expected, which is known as the ‘niche-breadth range-size hypothesis’ [3]. Over the last decades, many studies have tested this hypothesis, but supporting evidence remains debated (see e.g., [4] and references therein). Some argue that the correlation between niche breadth and range size is a statistical, rather than ecological effect (e.g., [5]), while others have claimed that the hypothesis represents a general ecological pattern [2]. This debate has important implications for ecology because the niche breadth-range size hypothesis is one of the mechanisms thought to explain ecological commonness and rarity [6]. In summary, species that decline in abundance usually occupy fewer sites, while those increasing in abundance tend to occupy more sites. Consequently, intraspecific abundance-occupancy relationships are typically positive. This relationship can be explained by temporal variations in resource availability, with species exhibiting greater niche breadth potentially showing more pronounced fluctuations in distribution and abundance compared to those utilising relatively invariant resources or capable of resource switching. Thus, the niche breadth-range size hypothesis offers a means to formulate additional testable hypotheses regarding the factors influencing the commonness and rarity within communities [7], necessitating to be put to the test across biological systems and evolutionary scales.

The niche breadth-range size hypothesis has been used to explain broad-scale macroevolutionary patterns. For instance, Janz and Nylin [8] used the niche breadth-range size hypothesis to explain the remarkable biodiversity of herbivorous insects, whereby cycles of dietary generalism associated with range expansions followed by local adaptations could explain the high biodiversity observed in this group. This is likely to be driven by factors associated with the utilisation of resources and the dietary niche, and not related to species’ dispersal ability, as shown in European butterflies (e.g., [9]; see also [10]). Botts et al. [11] highlighted a counterpart aspect of the niche breadth-range size hypothesis by showing that narrow niche breadth and the associated small range sizes are strong predictors of species’ likelihood of experiencing range contractions, which could translate into higher extinction risk. These studies exemplify the importance of the niche breadth-range size hypothesis to our understanding of population dynamics and the interactions between populations with their environment on both local and global scales [10].

Diet breadth (DBR) is a major factor influencing a species’ niche and its ability to utilise resources and expand geographic ranges [8]. As a result, dietary dimensions of the niche should also be considered alongside climatic niche dimensions in studies of the niche breadth-range size hypothesis. In herbivorous insects, a majority of species fall into the ‘specialism’ and few into the ‘generalism’ sides of the DBR spectrum [12] which contributes to the diversification within the group [13–15]. However, while evidence appears to support the diet niche breadth-range size hypothesis in herbivorous insects (e.g., [16–18]) the support for the hypothesis in non-herbivorous insects remains unclear. Blood-sucking (hematophagous) insects are interesting in this respect because of their ecological role in the spread of diseases to wild populations as well as their public health implications for disease transmission to humans and domesticated animals [19]. Moreover, the dependency of hematophagous insects on hosts could strengthen the relationship of niche breadth and range size with respect to dietary breadth, as species feeding on a wider range of hosts could occupy wider geographic areas without risks associated with host encounter rates. Thus, if the niche breadth-range size hypothesis is valid for hematophagous species, we should observe a positive relationship between diet breadth and range size, perhaps even stronger than the relationship between climatic niche breadth and range size. Krasnov et al. [20] tested this hypothesis in fleas and found some support for the dietary niche breadth-range size hypothesis, whereby more northern species had wider geographic ranges and also utilised more distinct and variable hosts (but note that the host number used was not northerly distributed). Having said that, fleas are a highly specialised order of insects, thus, the generality of their results and the support for the hypothesis in hematophagous insects remains to be investigated in other insect groups.

In this study, we tested the niche breadth-range size hypothesis in Neotropical Triatominae (Hemiptera: Reduviidae) using the most comprehensive dataset on their feeding patterns assembled to date. If the niche breadth-range size hypothesis is valid in its canonical form for hematophagous species, we predicted a positive relationship between climatic and diet niche breadths and range size. The Triatominae subfamily, commonly known as kissing bugs and cone-nose bugs, are almost exclusively blood-feeding true bugs and live in close association with their hosts [21]. Many triatomine species carry the parasite *Trypanosoma cruzi*, a flagellate protozoan that causes Chagas disease in animals and humans. The disease has been known to affect human populations since ancient civilization times, posing a long-standing public health threat to communities in the Global South. For instance, Guhl et al. [22], in their study of exhumed mummies from archaeological sites in Peru and Chile, established that human cases of Chagas’ disease could be traced back to 4000 B.C. Thus, a better understanding of how triatomine distributes in space and time as a function of environmental and dietary factors can provide invaluable insights into the ecology of kissing bugs, and their public health position [23,24]. Using our reconstructed dated phylogeny of Triatominae (see S1 Appendix), we first tested whether range size, climatic (i.e., temperature and precipitation) and dietary niche breadths displayed phylogenetic signals as a proxy for niche conservatism. Previous literature has shown that triatomine displays environmental niche conservatism [25]. Thus, triatomines are an ideal model to test whether the relationship between climatic and dietary niche breadth and geographic range size also shows conservatism. We then tested whether the predictions of the range size-niche breadth hypothesis were realised in our dataset. Overall, our study directly tests the niche breadth-range size hypothesis in an ecologically and public health important group of hematophagous insects, providing a better understanding of how blood-feeding triatomines occupy and interact with their environment.

## 2. Material and methods

### 2.1. Data collection

To characterise the range distribution of Triatominae species and their dietary and climatic niche, we conducted a comprehensive review of the literature as of 2021, compiling data from 158 published studies (S1 Table). We obtained data on feeding profiles and habitat use for four of the six tribes of the Triatominae (Rhodniini, Triatomini, Bolboderini, and Cavernicolini), including 9 genera (*Belminus*, *Cavernicola*, *Eratyrus*, *Mepraia*, *Panstrongylus*, *Paratriatoma*, *Psammolestes*, *Rhodnius*, and *Triatoma*). The primary information for the analysis was extracted from an exceptional and extensive Triatominae database [26]. We complemented the information from the database with a survey of over 500 bibliographic references between 1999 and 2021 obtained from the free access Chagas disease and triatomine bibliographic database BibTri (https://bibtri.cepave.edu.ar/), from which only 158 publications had data on blood-feeding profiles for 61 species of triatomines (S1 Table). From these, we obtained the blood-feeding patterns for species with reliable blood-feeding profiles sampled under natural conditions. As for habitat use, our dataset encompasses the habitats of epidemiological importance typically studied in Chagas disease vectors research: domiciliary, peridomiciliary, and sylvatic habitats [27].

We then added approximate geographic coordinates and obtained climatic niche variables by retrieving occurrence data from GBIF (www.gbif.org, accessed on 24 October 2023), pruning missing data, duplicates, and potentially misidentified occurrences. GBIF doi are available in Table 1. The environmental variables used to estimate the climatic niches were downloaded from the WorldClim v.2.1 database [28] at a spatial resolution of 10 arc-min (~18.5 km^2^) using the *worldclim_global* function in "geodata" v.0.5–9 R package [29]. This dataset includes 19 bioclimatic variables (bio1-bio19), such as temperature, precipitation, and seasonality. However, as we were interested in calculating the climatic niche breadth, we only used four variables from this dataset: maximum temperature of the warmest month (bio5), minimum temperature of the coldest month (bio6), precipitation of the wettest quarter (bio16), and precipitation of the driest quarter (bio17). The variables bio5 and bio6 were selected because they are usually the dominant thermal drivers for triatomines [30], while bio16 and bio17 were resorted to because precipitation also plays a decisive part in the distribution and the spatial delimitation of different triatomine species [25,31]. Collectively, our data allowed us to test directly the niche breadth-range size hypothesis in Triatominae.

**Table 1:** GBIF doi references from the respective species. GBIF doi references from the respective species. Data was accessed from R api via the ‘rgbif’ package (https://github.com/ropensci/rgbif) on 2024-06-12.

*Species*	GBIF doi
*Cavernicola pilosa*	https://doi.org/10.15468/dl.fgjmhe
*Mepraia spinolai*	https://doi.org/10.15468/dl.ykmd9a
*Panstrongylus chinai*	https://doi.org/10.15468/dl.dkyrdt
*Panstrongylus geniculatus*	https://doi.org/10.15468/dl.6ncf46
*Panstrongylus howardi*	https://doi.org/10.15468/dl.wegyqp
*Panstrongylus lignarius*	https://doi.org/10.15468/dl.e4453c
*Panstrongylus lutzi*	https://doi.org/10.15468/dl.fbqfny
*Panstrongylus megistus*	https://doi.org/10.15468/dl.rb5v5v
*Panstrongylus rufotuberculatus*	https://doi.org/10.15468/dl.yfan38
*Panstrongylus tibiamaculatus*	https://doi.org/10.15468/dl.gj6sjc
*Panstrongylus tupynambai*	https://doi.org/10.15468/dl.r7hkjp
*Paratriatoma hirsuta*	https://doi.org/10.15468/dl.ewy9aa
*Paratriatoma lecticularia*	https://doi.org/10.15468/dl.vykhsp
*Psammolestes coreodes*	https://doi.org/10.15468/dl.yvctbn
*Psammolestes tertius*	https://doi.org/10.15468/dl.nzer7w
*Rhodnius ecuadoriensis*	https://doi.org/10.15468/dl.u3xun3
*Rhodnius nasutus*	https://doi.org/10.15468/dl.5b3pzc
*Rhodnius neglectus*	https://doi.org/10.15468/dl.efvrsw
*Rhodnius pallescens*	https://doi.org/10.15468/dl.5rzbkx
*Rhodnius pictipes*	https://doi.org/10.15468/dl.59jmph
*Rhodnius prolixus*	https://doi.org/10.15468/dl.ms87fs
*Rhodnius robustus*	https://doi.org/10.15468/dl.krnfx7
*Triatoma barberi*	https://doi.org/10.15468/dl.z6jpmc
*Triatoma brasiliensis*	https://doi.org/10.15468/dl.yr8v5y
*Triatoma costalimai*	https://doi.org/10.15468/dl.k8vj4y
*Triatoma dimidiata*	https://doi.org/10.15468/dl.vwa2hu
*Triatoma eratyrusiformis*	https://doi.org/10.15468/dl.x99ttm
*Triatoma gerstaeckeri*	https://doi.org/10.15468/dl.4mva7a
*Triatoma indictiva*	https://doi.org/10.15468/dl.49nukp
*Triatoma infestans*	https://doi.org/10.15468/dl.nn9827
*Triatoma juazeirensis*	https://doi.org/10.15468/dl.wuv32h
*Triatoma longipennis*	https://doi.org/10.15468/dl.7mmrgb
*Triatoma maculata*	https://doi.org/10.15468/dl.u7y445
*Triatoma melanica*	https://doi.org/10.15468/dl.h5q7df
*Triatoma melanocephala*	https://doi.org/10.15468/dl.anu7nf
*Triatoma pallidipennis*	https://doi.org/10.15468/dl.9vdf5q
*Triatoma petrocchiae*	https://doi.org/10.15468/dl.uzxjjy
*Triatoma phyllosoma*	https://doi.org/10.15468/dl.rums92
*Triatoma platensis*	https://doi.org/10.15468/dl.4er3e4
*Triatoma protracta*	https://doi.org/10.15468/dl.jube3a
*Triatoma pseudomaculata*	https://doi.org/10.15468/dl.rs9y7g
*Triatoma recurva*	https://doi.org/10.15468/dl.qpaed6
*Triatoma rubida*	https://doi.org/10.15468/dl.gts88f
*Triatoma rubrofasciata*	https://doi.org/10.15468/dl.tkd66w
*Triatoma rubrovaria*	https://doi.org/10.15468/dl.w2dx5j
*Triatoma sanguisuga*	https://doi.org/10.15468/dl.fsta6x
*Triatoma sherlocki*	https://doi.org/10.15468/dl.bnma72
*Triatoma sordida*	https://doi.org/10.15468/dl.yep5vj
*Triatoma venosa*	https://doi.org/10.15468/dl.9nuxkw
*Triatoma vitticeps*	https://doi.org/10.15468/dl.4att3q

### 2.2. Data processing

Blood-feeding profiles were calculated as the number of collected insects that fed in one or more host species. When some triatomine individuals showed a mixed diet (i.e., multiple host species), they were distributed to the corresponding feeding category in the same proportion as that in which the individual feeding categories were found. An analysis of the feeding niche breadth of a set of 30 species was conducted in the past [27]. We updated this dataset by adding the most recent publications on the Triatominae diet (from the same database BibTri) that used recent biochemical and molecular methodologies to identify insect feeding strategies (S1 Table). These methodologies included (i) ELISA/IMM: Enzyme-linked immunosorbent assay (ELISA), immunoradiometric assay (IRMA), immunochromatographic assay, or immuno-double-diffusion (IMM), and (ii) DNA/PCR: recombinant cDNA techniques and Polymerase Chain Reaction (PCR). The addition of these bibliographic sources resulted in a dataset containing 61 triatomine species (S1 Table).

**Fig 1:**
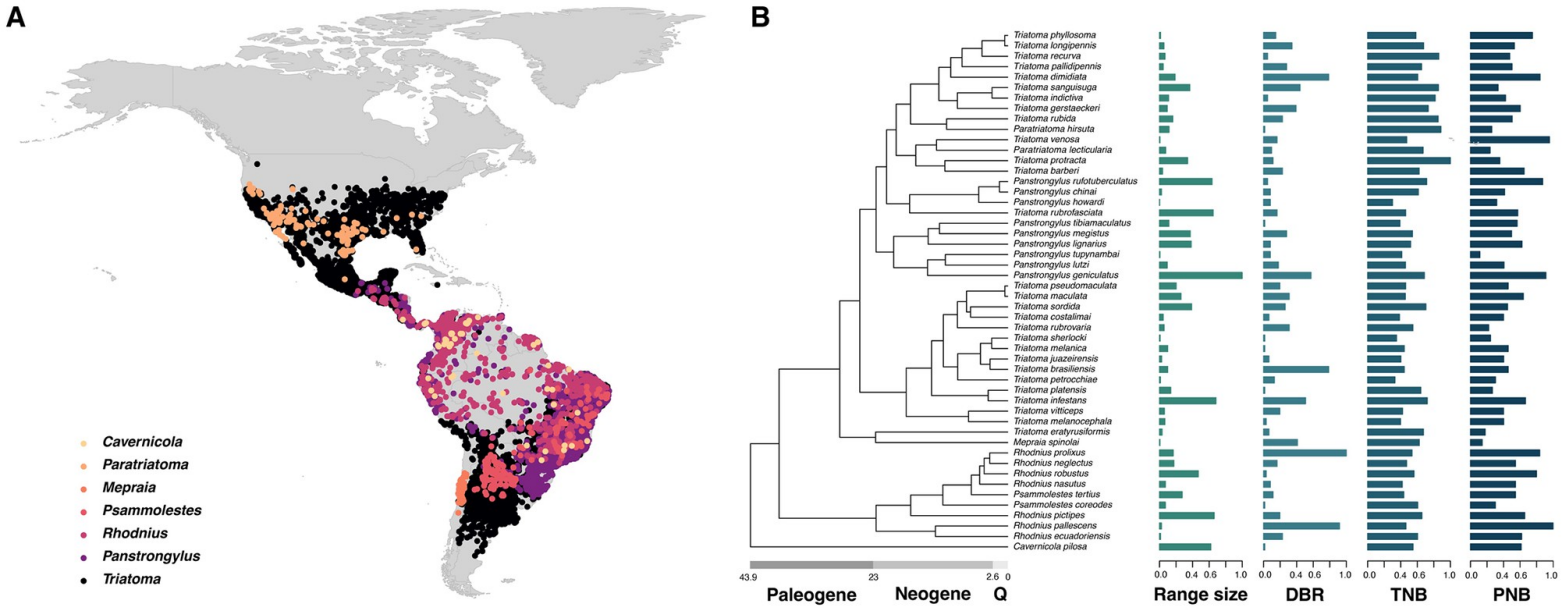
Overview of the hypotheses tested. (A). Occurrence points from each studied species are plotted. World country polygons were plotted using “rnaturalearth” v.1.0–1 R package [35]. (B). Phylogeny reconstructed in our study, with bar charts indicating the range size, species-level diet breadth, temperature niche breadth, and precipitation niche breadth. To facilitate visualisation, variables were scaled to 1. Coloured points at the tips of the phylogeny represent the occurrences of the species in (A). DBR = “Diet niche breadth”, TNB = “Temperature niche breadth”, PNB = “Precipitation niche breadth”.

To calculate the range size for each species, we performed alpha hull analyses in the occurrence points collected. We constrained the geographic distribution of our analysis to the Americas because species outside this region lacked diet data (Fig 1A). In total, there were only 38 out of 56,236 observations outside the Americas, distributed across China (2), Indonesia (14), India (9), Sri Lanka (4), Philippines (5), and Vietnam (4) for the entire period and across all species. Additionally, including these observations in the analysis could introduce biases in our estimates of climatic niche breadth, because they could artificially broaden the environmental ranges and suitable habitats of the species. Moreover, South America is the most likely origin for the Linshcosteus-*T*. *rubrofasciata* Asiatic clades of triatominae [32]. The alpha hull for each species was estimated using the *getDynamicAlphaHull* function from "rangeBuilder" v.2.1 R package [33]. The range size of the estimated alpha hulls was calculated with the *st_area* function in "sf" v.1.0–14 R package [34] and transformed to km^2^.

### 2.3. Diet and climatic niche breadth

Diet breadth (DBR) for each triatomine species was calculated as the number of unique taxonomic units used as feeding hosts; this was carried out at five host taxonomic levels: class (classDBR), order (orderDBR), family (familyDBR), genus (genusDBR), and species (speciesDBR) (S2 Table). Climatic niche breadth was estimated by extracting the climatic variables from the occurrence points of each species. The temperature niche breadth (TNB) was estimated through the temperature range in which the species was present, subtracting the lowest temperature from the minimum temperature of the coldest month (bio6) where the species was found from the highest value of the maximum temperature of the warmest month (bio5). We chose this temperature range given that it represents the impact of extreme temperatures across the year and the species range [36]. Indeed, Moo-Llanes et al. [37] showed that the geographical distribution of triatomine species is greatly influenced by temperature seasonality. For the precipitation niche breadth (PNB), we calculated the range as the difference between the minimum value of driest quarter precipitation (bio17) and the maximum value of wettest quarter precipitation (bio16) over the geographic distribution of each species. In this case, the quarter precipitation values could better reflect the occurrence of species across the year [38].

### 2.4. Data analysis

In order to assess whether the niche breadth is influenced by the shared evolutionary history of a species [39], we calculated the phylogenetic signal of TNB, PNB, DBR, and range size. It is important to assess the effect of the phylogeny in the evolution of the characters across species, as this would indicate other factors, such as the degree of phylogenetic relatedness among species, affecting the results. The phylogenetic signal measures the degree to which a trait evolves according to a Brownian motion model, in which high values indicate that related species tend to be more similar than random species. These analyses were conducted using two indices: Pagel’s λ [40] and Blomberg’s K [41], the latter being more conservative. Both indices vary between 0 and 1, with 0 indicating no phylogenetic structure in the data, whereas 1 indicates phylogenetic structure congruent with the expected under the Brownian motion model of evolution. These calculations were made using the *phyloSignal* function from the “phylosignal” R package, v.1.3 [42], using 1,000 simulations for the estimation of p-values. The phylogeny used in the analyses was reconstructed for all species for which ecological and dietary data was available (see S1 Appendix for the phylogeny reconstruction). The analyses were repeated for 1,000 alternative topologies from the posterior distribution.

Finally, to test the niche breadth-range size hypothesis, we performed simple multivariate regressions and Phylogenetic Generalized Least Squares (PGLS) analyses. The multivariate regressions were performed given that the phylogenetic signal did not recover any statistically significant result (see Results). However, we also performed the PGLS estimating λ with maximum likelihood to allow for this lack of phylogenetic signal. Before fitting the regressions, we assessed the collinearity between the predictor variables (i.e., DBR, TNB, and PNB) using the “correlation” v.0.8.3 R package [43], but no predictor presented a high level of correlation (r < 0.4). Then, using the *lm* function [44] and *pgls* function from the “caper” R package, v.1.0.1 [45], the model fitted was:

Rangesize~Dietbreadth+Temperaturenichebreadth+Precipitationnichebreadth

This model was run across all five taxonomic host levels of the diet breadth. A log transformation was applied to range size and diet breadth before the analyses, while a square root transformation was applied to temperature and precipitation niche breadth. In addition to this fitted model for all species in the dataset, we also incorporated in the analyses the habitat type of each species, due to the influences that habitat can have on the kissing bug feeding choices [27]. Therefore, we replicated all analyses for each habitat type, that is, domiciliary, peridomiciliary, and sylvatic. The habitat type was not used as a predictor, considering that most species are present in more than one habitat. We repeated the PGLS analyses for 1,000 alternative topologies. All analyses were performed on R 4.2.2 [44].

## 3. Results

### 3.1. No evidence of niche conservatism in triatominae

**Fig 2:**
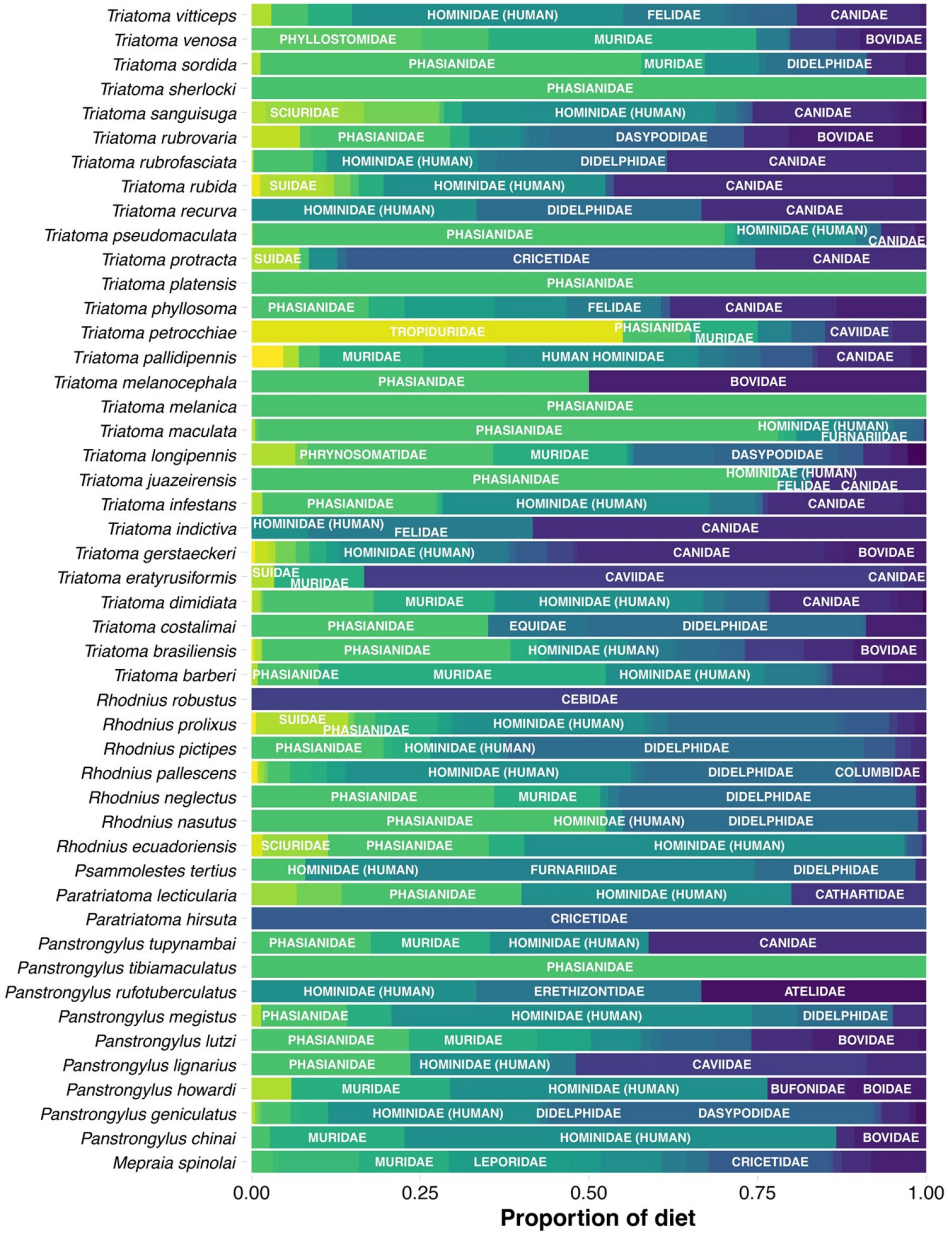
Proportions of each host family used as diet by each triatomine species. Only the names of the three families with the higher proportions are shown. Colours correspond to the host families used by each triatomine.

We first qualitatively investigated the proportion of host families used by each triatomine species to gain insights into the dietary diversity of this blood-feeding group. Hominidae (human), Phasianidae, and Canidae families were among the most common items in the diet of these species (Fig 2). More specifically, proportions of Hominidae (human) in triatomine’s diet ranged from 0.734% for *Triatoma longipennis* to 100% for *T*. *carrioni*, proportions of Phasianidae ranged from 0.678% for *T*. *sanguisuga* to 100% for *T*. *lenti*, *T*. *melanica*, *T*. *platensis*, *T*. *sherlocki*, *Panstrongylus tibiamaculatus*, and proportions of Canidae ranged from 0.015% for *T*. *maculata* to 58.33% for *T*. *indictiva*. This shows that triatomine displays a remarkable variability in host species use.

We then tested whether range size, temperature, precipitation, and diet niche breadths displayed any phylogenetic signal as a sign of niche conservatism [46]. None of our measurements of range size and dietary or climatic niche breadths showed evidence of niche conservatism (Table 2; Fig 1B). Together these results indicate that dietary and climatic niches evolve in a lineage-specific way and independent of shared evolutionary histories in the Triatominae.

**Table 2:** Phylogenetic signal analysis results for the continuous variables studied here.

Trait	K	p-value	λ	p-value
**Area**	0.186 (0.124–0.258)	0.393 (0.209–0.62)	0 (0–0)	1 (1–1)
**Temperature niche breadth**	0.145 (0.053–0.212)	0.605 (0.269–0.906)	0 (0–0)	1 (1–1)
**Precipitation niche breadth**	0.115 (0.035–0.178)	0.79 (0.497–0.982)	0 (0–0)	1 (1–1)
**Diet niche breadth: species**	0.203 (0.108–0.288)	0.346 (0.174–0.635)	0.054 (0–0.193)	0.917 (0.419–1)
**Diet niche breadth: genus**	0.206 (0.109–0.288)	0.317 (0.168–0.603)	0 (0–0.172)	1 (0.45–1)
**Diet niche breadth: family**	0.188 (0.094–0.263)	0.398 (0.221–0.711)	0 (0–0.046)	1 (0.765–1)
**Diet niche breadth: order**	0.163 (0.073–0.238)	0.48 (0.215–0.844)	0 (0–0)	1 (1–1)
**Diet niche breadth: class**	0.134 (0.053–0.191)	0.659 (0.407–0.971)	0 (0–0)	1 (1–1)

The value refers to the median value across topologies, with 95% confidence intervals enclosed in parenthesis.

### 3.2. Supporting evidence for the range size-niche breadth hypothesis for climatic but not dietary niche breadths

**Fig 3:**
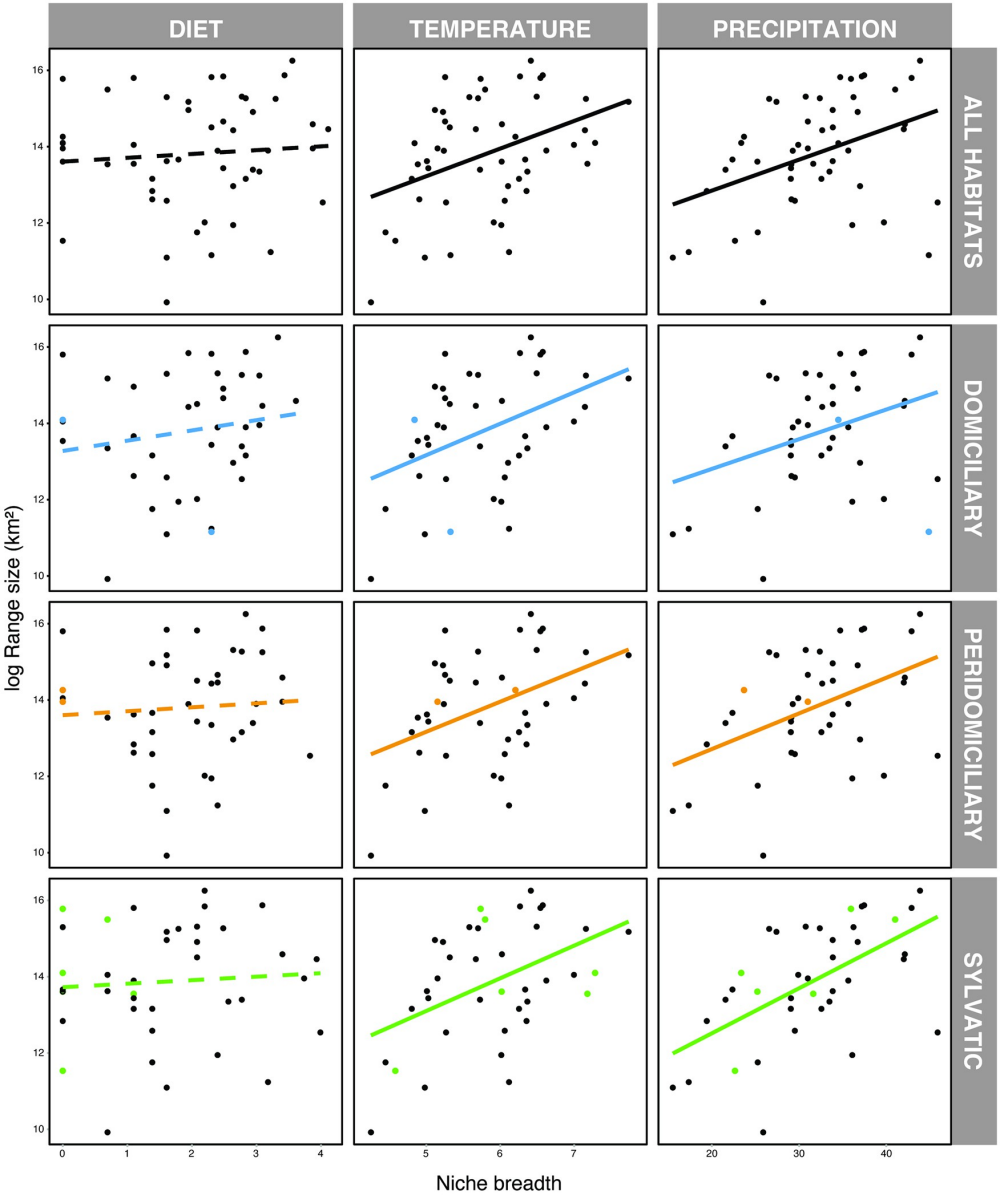
Relationships between the predictor and response variables analysed using diet breadth at the species level. The natural log of the range size was used as the response in a multivariate regression, whereas the natural log of the diet breadth (first column) and square root transformed temperature (second column) and precipitation (third column) niche breadths were used as predictors. Each row corresponds to the analyses for the entire dataset and divided by triatomines’ habitat. Linear trend lines were added to facilitate interpretation according to the intercept and slope from the regression calculations. Dashed lines represent non-significant relationships, whereas solid lines are statistically significant. Coloured points correspond to the species exclusive of the respective habitat.

Next, we tested the predictions for the range size-niche breadth hypothesis in Triatominae. This was done for the entire dataset as well as for each habitat in which species were encountered separately (i.e., sylvatic, peridomiciliary, domiciliary). Our rationale was that habitat could influence the relationship between dietary and climatic niche breadth and range size. It is important to note that the majority of species occurred in more than one habitat (domiciliary = 4%; peridomiciliary = 4%; sylvatic = 12%; domiciliary and peridomiciliary = 16%; domiciliary and sylvatic = 2%; peridomiciliary and sylvatic = 2%; and domiciliary, peridomiciliary, and sylvatic = 60%). Our results showed no relationship between diet breadth and range size in either the entire dataset or within each habitat type (first column in Fig 3; S3 Table). Both temperature and precipitation niche breadths were positively associated with range size, even in the analyses considering each habitat separately (second and third columns in Fig 3; S3 Table). These results were consistent across all host taxonomic levels of diet breadth (i.e., from species to class; S3 Table) and in the phylogenetically controlled linear regression models (S4 Table), which was expected since none of the niche breadth variables showed evidence of phylogenetic signal (see above). These results provide support for the predictions of the range size-diet breadth hypothesis concerning temperature and precipitation niche breadths but not in relation to diet breadth in Triatominae.

## 4. Discussion

Ecological theory predicts that species that can utilise a greater array of resources (‘niche breadth’) should occupy larger geographical ranges [1,2]. We tested the predictions of this hypothesis in a group of species of ecological and public health interests, the Neotropical Triatominae, vectors of the Chagas disease in the Americas. Our results revealed that temperature and precipitation niche breadths were positively correlated with range sizes independent of the habitat type, whereas diet breadth was not. These findings provide supporting evidence for the range size-niche breadth hypothesis for the climatic niche axis of the multidimensional niche, and highlight that not all niche axes correlate in the same manner with range size, which can explain the inconsistent evidence supporting the range size-niche breadth hypothesis across taxa [2,9,18,47].

Our results showed no evidence of niche conservatism in Triatominae. This is in broad agreement with the Triatominae literature (e.g., [48–50]), although it contradicts some previous findings. For instance, Ibarra-Cerdeña et al. [25] found evidence for climatic niche conservatism in Triatominae, but our data does not provide the same level of evidence to support such findings. There are two potential reasons for this difference. Firstly, triatomines at higher latitudes tend to be more closely related species and share similar niche preferences [48], the reason why Ibarra-Cerdeña et al. [25] results report niche conservatism for species distributed in Central and North America, while we found weaker effects, due to our more complete dataset with Neotropical species. This argument is supported by recent studies that did not find support for niche conservatism in Neotropical lineages of triatomines [49,50]. A second potential reason for the disparity between our results and that of Ibarra-Cerdeña et al. [25] could be attributed to differences in data processing and methodology. Ibarra-Cerdeña et al. [25] also used Schoener’s *D* to quantify niche overlap between two species over the environmental space, while we calculated a phylogenetic signal that considers the entire range of species in the phylogeny rather than niche overlap. Furthermore, the species’ divergence estimates also coincide with previous studies [51,52] and support the rapid and ample diversification of Triatominae, which started in the Miocene and was influenced by ecological and environmental changes resulting from the Andean uplift [53]. Therefore, our results are in broad agreement with recent findings in the Triatominae literature.

Diet breadth was not correlated with range sizes in Triatominae in our study, suggesting that Triatominae’s ability to explore different host species is independent of its habitat type or geographic range. These results add to the debate on the support of the niche breadth-range size hypothesis in the literature. This is because our results show that some, but not all, niche breadth variables are correlated with range sizes and one has to exercise judgment as to how to select and interpret the (lack of) relationship between niche breadth and range size. One possible explanation for the lack of association between diet breadth and range size concerns the host of triatomes. Some species may have a narrow range of hosts, but these hosts have large distributions. This can lead to large range sizes for species with low and high diet breadth, affecting the possible positive relationship between these variables. Currently, we lack high-resolution data to address this for the 90 host families used by the Triatominae in this study, but this remains a primary area for future investigations. In contrast, the significant correlation found between range size and climatic niche could emerge because many Triatominae feed on small mammals [54]. These small mammals are sensitive to rainfall oscillations because it dictates the productivity of their food sources. This, in turn, affects the population dynamics of small mammals and could lead to effects on other species in the trophic web (including the Triatominae) [55–57], Small mammals are only a few among the many hosts of the Triatominae, and this could explain why the relationship between precipitation and range size is statistically significant in our analysis but not the relationship between diet breadth and range size (i.e. not all hosts rely on the precipitation regimen for food sources).

The debate in the literature is far from a definitive answer for the niche breadth-range size hypothesis in triatomines. Slatyer et al. [2] conducted a meta-analysis using 64 studies that measured niche breadth and range size of plant species and found a significant positive relationship between range size and environmental tolerance breadth, habitat breadth, and diet breadth, but with significant variability in the strength of the relationship among studies. Kambach et al. [4] found that a species’ realised niche breadth estimated at the regional level is a weak predictor of a species’ global niche breadth and range size. Previous studies have gone so far as to suggest that a positive relationship between niche breadth and range sizes can emerge as statistical artefacts. For instance, Ficetola et al. [58] raised the possibility that correlations between niche breadth and range size can emerge as a by-product of the strong spatial structure of environmental variables. This is because they showed that fine-scale data of microhabitats provided a more direct measure of conditions selected by ectotherms, particularly European plethodontid salamander species of the genus *Hydromantes*, which enabled more accurate estimates of niche breadth in this group. Likewise, Saupe et al. [59] tested two propositions using a computational approach: (a) strong geographical patterns in realised niche breadth variation can arise in the absence of variance in fundamental niche breadth size, and (b) realised niche breadths can show latitudinal patterns as a consequence of spatiotemporal climate change, even when fundamental niche breadths are unrelated to latitude and dispersal abilities are held constant. They concluded that tropical species can have narrower niche breadths for maximum and minimum temperature ranges compared with temperate species solely as the result of the spatial arrangement of environments and that the complex spatiotemporal distribution of global abiotic environments has a strong potential for structuring observed latitudinal gradients of niche breadths. Moore et al. [5], using phylogenetic least squares regression, examined the extent to which variation in range size of *Pelargonium* species (plants of the family Geraniaceae) is related to temperature and precipitation niche breadths, and tested whether observed niche breadth-range size relationships were stronger than expected, given spatial autocorrelation of climatic variables. They concluded that spatial autocorrelation may positively bias niche breadth-range size relationships. This bias suggests that previously reported relationships between range size and niche breadth based on broad-scale distributional data may be, at least in part, artefactual. Similar results were presented by Morimoto [18] in Sydney butterflies, showing that the relationship between niche breadth and range size may emerge as a product of the scale upon which niche breadth is estimated.

The interaction of Triatominae species with their environment sheds light on how climate change influences their range sizes, diet breadth, and potential impact on public health. Species with wider range sizes often have broader niche breadths in terms of diet or climate tolerance, such as *Rhodnius prolixus*, an important vector of Chagas disease. As temperatures rise due to climate change, these vectors are likely to expand their range, exacerbating Chagas disease’s public health issues. Studies have forecasted significant increases in Chagas disease prevalence and economic burden under climate change scenarios. For example, Tamayo et al. [60] found that higher temperatures accelerate the life cycle and increase the fecundity and infective forms of *R*. *prolixus*, suggesting a higher likelihood of Chagas disease transmission. Some studies have suggested a reduction in Triatominae distribution (e.g. [61–62]) but Belliard et al. [63] found that acclimation positively affects thermotolerance, indicating triatomine species can adapt to temperature variations and potentially disperse more widely. Our findings emphasise the relationship between range size and climatic and diet breadth, indicating that species with wider diet breadths, particularly those including humans as hosts, could become increasingly problematic in urban or peri-urban areas, exposing populations to increasing risks of Chagas disease. This is likely to affect deprived areas of the population as Chagas disease is disproportionately more prevalent and more likely to occur in these areas, like many other neglected tropical diseases [64–65]. Raising awareness of these patterns can help us achieve our sustainable development goals and support the socio-economic development in Latin America [66].

Some interesting potential relationships are beyond the scope of the present study but are attractive open questions for future research, such as the possible relationship between niche breadth and, e.g., (a) proximity to range boundaries [67]; (b) body size [47]; (c) latitude [68]; (d) diversity and competition [47]; (e) habitat fragmentation [67]; and (f) the evolution of niche breadth and plasticity [69]. Another factor that might explain our results is the dominant proportion of species that utilise domiciliary/peri-domiciliary habitat types in the collated dataset (0.88) compared with the species of surely sylvatic or sylvatic combined with domiciliary/peri-domiciliary habitat types (0.12). The exclusively domiciliary/peri-domiciliary habitat types share many of the same dominant hosts (humans, dogs, chickens, and cats) which are somewhat concentrated in (peri-)urban areas, which could have contributed to the absence of a relationship between diet niche breadth and geographical range size, as well as the niche conservatism. Nevertheless, at a macroecological scale, our results show that the validity of the niche breadth-range size hypothesis holds only for temperature and precipitation niche breadth but not dietary niche breadth in this group of hematophagous species.

As with other studies using species occurrences from databases and literature data (e.g. [25,49]), our assessment of the relationship between diet breadth and range size can have influences of sampling biases. For instance, it is known that some regions of isolated pristine areas in the neotropics, such as parts of the Amazon Forest, are under-sampled. This can lead to an underestimation of species range size, climatic, and diet breadth. We minimized these biases by comprehensively assessing the literature and the species occurrence database to ensure that our inferences were conducted to the best available information about the Triatominae. Still, the occurrences of the species studied may not cover their entire known range. Therefore, a literature review approach may prove useful for gathering data for future studies and may increase the accuracy of climatic niche estimation. Moreover, our estimates of diet breadth come from studies using ELISA and PCR, which are highly sensitive techniques with extremely high resolution of hosts. It is also important to mention that the evolutionary relationships between species used here were inferred using the most recent genetic data available. More work is needed to advance the systematics and phylogenetics of the Triatominae. Still, our work has used the most reliable estimate of evolutionary relationships possible from the genetic data. Future studies and observations using e.g. drones [70–71], which can sample pristine regions, will expand our knowledge of the distribution and diet breadth of the Triatominae in isolated areas, as well as their genetic architecture, and possibly, lead to the discovery of new species. This will strengthen the robustness of future applications of our approaches to Triatominae and broaden the spectrum of species encompassed in niche breadth analyses in other species. Finally, it will be important for an alternate approach correlating the characteristics of the microclimate and the macroclimate of the Triatominae species. This will allow us to ensure that our findings at a macroecological scale match that of a local or regional scale.

## 5. Conclusion

We tested the niche breadth-range size hypothesis in the subfamily Triatominae, a group of blood-feeding true bugs that are vectors for Chagas disease, one of the most important neglected tropical diseases in Latin America. We found that the climatic niche breadth, represented as temperature and precipitation, but not the dietary niche, is associated with range sizes in Triatominae. Moreover, consistent with prior literature findings, there was no evidence of niche conservatism detected in Triatominae, given their rapid and ample diversification uncovered by our species’ divergence estimates. With the increasing availability of species distribution data, it will be important for future studies to integrate the distribution range of the hundreds of hosts into the distribution of triatomine species. This will likely refine our understanding of how each species is distributed along environmental gradients. In the present study, we gained macroecological insight into the relationship between diet breadth and range size, testing a well-established ecological hypothesis in this group of blood-feeding insects. Overall, our results have implications for better understanding the ecology and biogeography of this and other blood-feeding insect vectors, providing insights that can be used to predict, manage, and control the occurrence of vector-borne diseases.

## Supporting information

S1 Appendix Phylogenetic reconstruction. (DOCX)

S1 Dataset Genetic sequences used for phylogenetic reconstruction. (ZIP)

S1 Table Publications used as references to retrieved triatomines diet data. (XLSX)

S2 Table Niche breadth, range size, and habitat data for each species used in this study. (XLSX)

S3 Table Summary of results from the ordinary least square regression of log(area) and log(diet), sqrt(temperature), and sqrt(precipitation) niche breadth. (XLSX)

S4 Table Summary of results from the PGLS analysis fitting the regression of log(area) and log(diet), sqrt(temperature), and sqrt(precipitation) niche breadth. (XLSX)

S5 Table Divergence times (Myr) estimated of Triatominae species based on secondary calibration points. (XLSX)
