# Supplementary material for: Range size positively correlates with temperature and precipitation niche breadths but not with dietary niche breadth in triatomine insects, vectors of Chagas disease
Source: PLoS Negl Trop Dis. 2024 Aug 16;18(8):e0012430. doi: 10.1371/journal.pntd.0012430 (PMC11357105; doi:10.1371/journal.pntd.0012430)
Supplement: S1 Appendix — (DOCX) [file pntd.0012430.s001.docx]

**S1 APPENDIX**

1. **Materials and methods**

## *Phylogenetic reconstruction*

A calibrated phylogeny of 50 triatomine species was constructed using sequences of three mitochondrial genes (12S rRNA, 16S rRNA, and cyt-b), available in the GenBank database (raw sequences in S1 Dataset). A lack of genetic data forced nine species out of the initial 59 to be pruned from the subsequent analyses. Cyt-b sequences were translated into amino acids to check for stop codons in Mesquite v.3.5 [1]. The alignment for each gene was performed in Geneious Prime with MAFFT v.7 [2] using auto strategy, and then concatenated. The resulting alignment had a total length of 2,448 base pairs (bp). Phylogenetic reconstruction was performed in BEAST v.2.6.6 [3] through the CIPRES portal (http://www.phylo.org). We built an ultrametric tree using a relaxed molecular clock method with uncorrelated lognormal rates among branches [4], assuming a Yule speciation tree prior [5], and with the GTR+G+I substitution model for the three genes. The tree calibration was made through two secondary calibration points for the Triatomini and Rhodniini tribes, with a normal distribution for the priors, following Hwang and Weirauch [6]. More specifically, we assigned mean priors of 31.26 Myr (SD = 3.5) for Triatomini and 22.18 Myr (SD = 3.2) for Rhodniini. The analyses were run for 6 x 10^8^ generations, sampling every 1,000 generations for two independent runs using different seed numbers. Tracer v.1.6 [7] was used to assess the convergence of chains, stationarity of the Markov chain Monte Carlo (MCMC) analyses, and effective sample size (>200) for each parameter estimated. We discarded a burn-in of 10% from the results, and the maximum clade credibility (MCC) tree was summarized with median node heights using TreeAnnotator v.2.6.6 [3]. We also randomly selected 1,000 pseudo-posterior samples to use in posterior analyses to account for phylogenetic uncertainty.

1. **Results**

Phylogenetic reconstructions of triatomine relationships reveal that the Triatominae lineage experienced its initial split during the Middle Eocene, approximately 39.06 Myr ago (95% C.I. = 27.8–53.5 Myr; S5 Table) into two clades (Fig 1B). This first divergence event gave rise to two lineages within Triatominae, consisting of the genera (1) *Triatoma*, *Mepraia*, *Panstrongylus*, and *Paratriatoma*, which diverged in 28.7 Myr (95% C.I. = 20.22–36.88 Myr; S5 Table), and (2) *Rhodnius* and *Psammolestes*, which diverged around 22.49 Myr (95% C.I. = 15.81–29.21 Myr; S5 Table). During the evolutionary history of Triatominae, the Neogene period (5.3–23 Myr) stands out as the time of significant speciation events (5.3–23 Myr; Fig 1B; S5 Table).

1. **References**

1. Maddison WP, Maddison DR. Mesquite: A modular system for evolutionary analysis. http://mesquiteproject.org. 2018.

2. Katoh K, Standley DM. MAFFT Multiple Sequence Alignment Software Version 7: Improvements in Performance and Usability. Mol Biol Evol. 2013;30(4):772–80.

3. Bouckaert R, Vaughan TG, Barido-Sottani J, Duchêne S, Fourment M, Gavryushkina A, et al. BEAST 2.5: An advanced software platform for Bayesian evolutionary analysis. PLOS Comput Biol. 2019;15(4):e1006650.

4. Drummond AJ, Ho SYW, Phillips MJ, Rambaut A. Relaxed Phylogenetics and Dating with Confidence. PLoS Biol. 2006;4(5):e88.

5. Bouckaert R, Heled J, Kühnert D, Vaughan T, Wu CH, Xie D, et al. BEAST 2: A Software Platform for Bayesian Evolutionary Analysis. PLoS Comput Biol. 2014;10(4):e1003537.

6. Hwang WS, Weirauch C. Evolutionary History of Assassin Bugs (Insecta: Hemiptera: Reduviidae): Insights from Divergence Dating and Ancestral State Reconstruction. PLoS ONE. 2012;7(9):e45523.

7. Rambaut A, Drummond AJ. Tracer v1. 6 http://beast.bio.ed.ac.uk. 2007.
